# Multifunctional Plasmonic Grating Based on the Phase Modulation of Excitation Light

**DOI:** 10.3390/nano11112941

**Published:** 2021-11-03

**Authors:** Sen Wang, Jing Zhang, Maixia Fu, Jingwen He, Xing Li

**Affiliations:** 1 Shandong Provincial Engineering and Technical Center of Light Manipulations & Shandong Provincial Key Laboratory of Optics and Photonic Device, College of Physics and Electronics, Shandong Normal University, Jinan 250014, China; zjjjzz202010@163.com; 2 Key Laboratory of Grain Information Processing and Control, College of Information Science and Engineering, Henan University of Technology, Zhengzhou 450001, China; fumaixia@126.com; 3 State Key Laboratory of Space-Ground Integrated Information Technology, Beijing Institute of Satellite Information Engineering, Beijing 100095, China; hejingwen880112@126.com

**Keywords:** surface plasmon polaritons, phase modulation, nondiffracting beam, focusing

## Abstract

Multifunctional optical devices are desirable at all times due to their features of flexibility and high efficiency. Based on the principle that the phase of excitation light can be transferred to the generated surface plasmon polaritons (SPPs), a plasmonic grating with three functions is proposed and numerically demonstrated. The Cherenkov SPPs wake or nondiffracting SPPs Bessel beam or focusing SPPs field can be correspondingly excited for the excitation light, which is modulated by a linear gradient phase or a symmetrical phase or a spherical phase, respectively. Moreover, the features of these functions such as the propagation direction of SPPs wake, the size and direction of the SPPs Bessel beam, and the position of SPPs focus can be dynamically manipulated. In consideration of the fact that no extra fabrication is required to obtain the different SPPs fields, the proposed approach can effectively reduce the cost in practical applications.

## 1. Introduction

Similar to an electromagnetic wave propagating along a metal/dielectric interface, surface plasmon polaritons (SPPs) are capable of shorter wavelength, tighter field confinement, and stronger field enhancement than the excitation light in the free space [1]. Owing to these unique properties, applications based on SPPs have been extensively exploited, ranging from plasmonic circuits [2], super resolution imaging [3], biosensing [4], and optical tweezers [5] to energy harvesting [6] and metamaterials [7]. The foundation of the aforementioned applications is through the modulation of the propagation and distribution of SPPs field. Therefore, the SPPs devices with different functions including the focusing lens [8,9], reflection mirror [10], hologram [11], logic operation [12], vortex [13,14], and nondiffracting beam generation [15,16,17] have been demonstrated by designing the position and shape of metallic or dielectric structures.

Traditional SPPs are usually static. Various methods were utilized to dynamically modulate the function of SPPs devices [18,19,20,21,22]. In 2011, the wavelength-multiplexed SPPs focusing field was demonstrated with a nonperiodic nanoslit array, which was designed by an iterative algorithm [18]. Using a nanohole array, Bergin Gjonaj et al. actively controlled the position of SPPs focus by modulating the amplitude and phase of incident beam [19]. In 2008, Erez Hasman et al. observed the polarization-dependent shift of SPPs focus generated by a semicircular plasmonic lens and explained this effect with the spin-orbit coupling [21,22]. Since then, the polarization-based dynamical SPPs focusing field [23,24,25], vortex [13,14], hologram [26,27], and nondiffracting beam [16,28] have been achieved. In addition, the nonlinear light-matter interaction [29], laser-induced thermal effect [30], and 2D materials such as graphene [31] were employed to manipulate the SPPs field. Nevertheless, different functions require the fabrication of the corresponding structure. The active modulations of SPPs fields are generally restricted to a single function. Although the multifunctional metasurface modulation of the transmission light (far field) is demonstrated [32,33], the near field SPPs devices with multiple functions are rarely discussed.

In this paper, through the modulation of the phase of excitation light, three different SPPs fields can be generated using a plasmonic grating. Simulations based on finite difference time domain (FDTD) method verified the feasibility of the proposed approach. The Cherenkov SPPs wake or nondiffracting SPPs Bessel beam or focusing SPPs field can be generated when the excitation light is imprinted with a linear gradient phase or a symmetrical phase or a spherical phase, respectively. The propagation direction of SPPs wake, the nondiffracting and self-healing properties of SPPs Bessel beam, and the position of SPPs focal point are analyzed. In addition, the relationship between each function and the corresponding phase is discussed. The proposed multifunctional plasmonic grating can play different roles as needed, which are efficiency and flexibility in applications.

## 2. Results and Discussion

**Figure 1:**
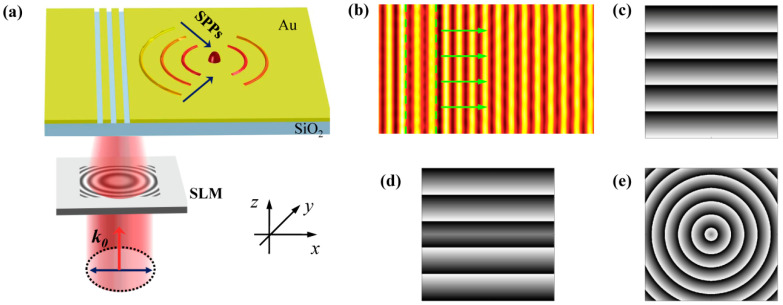
(**a**) Schematic diagram of the multifunctional plasmonic grating lens enabled by modulating the phase of light with SLM. (**b**) The SPPs wave propagates perpendicularly to the grating when no phase mask is loaded on the SLM. (**c**–**e**) The linear gradient phase, symmetrical phase, and spherical phase distributions addressed on the SLM, which can correspondingly excite the Cherenkov SPPs wake, nondiffracting SPPs Bessel beam, and focusing SPPs field.

Figure 1a schematically shows how the plasmonic grating realizes multiple functions. The plasmonic grating consists of subwavelength periodical slits etched on the gold film. A linearly polarized plane wave is normally incident on the spatial light modulator (SLM) and the wavefront of the transmitted wave is modulated by the phase mask loaded on the SLM. Then, the excitation light impinges on the plasmonic grating and the generated SPPs wave propagates along the gold/air interface. The phase mask carried by the excitation beam can be transferred to the SPPs wave. Therefore, by changing the phase mask addressed on the SLM, the wavefront of the SPPs wave is subsequently modulated and different functions can be accomplished. As shown in Figure 1b, without the phase mask, the SPPs field excited by the normally incident plane wave propagates perpendicularly to the grating, which is the fundamental function of a plasmonic grating. When a linear gradient phase in Figure 1c or a symmetrical phase in Figure 1d or a spherical phase in Figure 1e is loaded on the SLM, the Cherenkov SPPs wake [34], nondiffracting SPPs Bessel beam [28] or focusing SPPs field [8] can be correspondingly excited. Moreover, to obtain these phase modulations, the expensive SLM can be replaced by the optical wedge, axicon, and lens. Numerical simulations based on the finite difference time domain method (Lumerical FDTD Solutions) are conducted to analyze the various SPPs fields. In the simulations, the excitation light with a wavelength of 632.8 nm illuminates the plasmonic grating from the bottom. To obtain the optimum excitation of SPPs, the polarization is perpendicular to the grating since the transverse electric (TE) polarized light cannot excite the SPPs and only the transverse magnetic (TM) polarized light can give rise to SPPs [35,36]. The different phase modulations are generated by the script file editor and then imported into a simulation model. The boundary conditions in three directions are all set as perfect matched layers to avoid the reflection of the electromagnetic wave. The SPPs distributions are extracted from the frequency-domain field profile monitor. The thickness of the Au film usually ranges from 100 to 200 nm for the 632.8 nm excitation light [8,24,37] in the experiments. Therefore, the thickness of the Au film is chosen to be 150 nm, which can effectively avoid the transmission of excitation light. The substrate is set as SiO_2_, which is commonly used in plasmonic devices [8,24,29]. It is transparent for the 632.8 nm incident light and can support the Au film. The permittivity of the gold film is $\varepsilon_{m}=-11.82+1.24i$ and the wavelength of the excited SPPs $\lambda_{sp}$ is 606 nm, according to the dispersion curve of SPPs. The width of the slit is 150 nm and the period of grating is equal to the wavelength of SPPs, in order for the SPPs wave generated by each slit to interfere constructively. In the following sections, we analyze the SPPs fields generated by the excitation light and imprinted with the linear gradient phase, symmetrical phase, and spherical phase.

### 2.1. Cherenkov SPPs Wake

Initially, we modulate the excitation light with a linear gradient phase in Figure 1c, which can be expressed as follows:

(1) $$\varphi \left(x,y\right)=-k_{0}y\sin\alpha .$$

**Figure 2:**
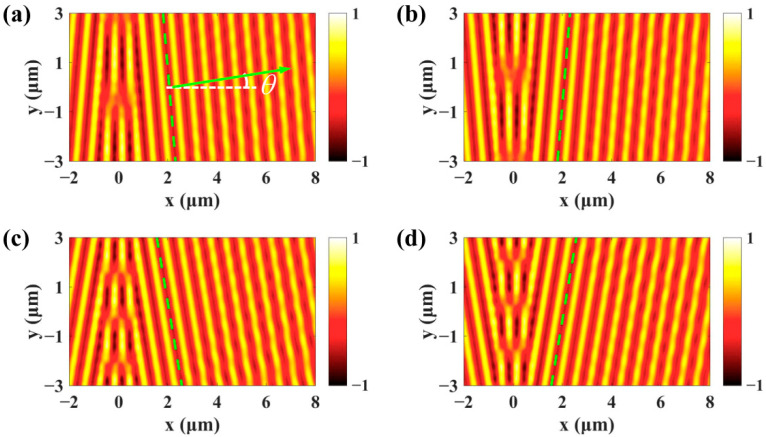
The propagation direction of the SPPs wave can be dynamically manipulated by changing the phase mask loaded on the SLM. (**a**–**d**) The real parts of SPPs field excited by the $\alpha =5^{\circ }$, $\alpha =-5^{\circ }$, $\alpha =10^{\circ }$, and $\alpha =-10^{\circ }$ incident light, respectively.

$k_{0}$ is the wave vector and α determines the incident angle of excitation light after transmission through the SLM. The grating can be divided into a series of subwavelength slits along the y direction and each subwavelength slit is regarded as a SPPs dipole [37]. For normal incidence light $\alpha =0^{\circ }$, all of the excited dipoles are in the phase and the SPPs plane wave propagates perpendicularly to the grating. For oblique incidence light, the gradient phase along the grating makes the SPPs plane wave propagate obliquely, which is also referred to as the Cherenkov SPPs wake [34]. As shown in Figure 2a, the propagation direction of SPPs wave is described by the angle θ and the expression is given by:

(2) $$\sin\theta =\frac{\sin\alpha }{n_{eff}},$$

where $n_{eff}=k_{0}/k_{sp}=\lambda_{sp}/\lambda_{0}$ is the effective index of the SPPs. The simulated real parts of SPPs fields generated by the $\alpha =5^{\circ }$ and $\alpha =-5^{\circ }$ incident light are presented in Figure 2a,b. It can be seen that the wavefront of SPPs wave denoted by the dashed green lines is tilted and the SPPs wave propagates upward and downward, respectively. The simulated propagation angle is $\theta =4.84^{\circ }$, which is in good consistency with the theoretical value $\theta =4.78^{\circ }$ obtained with Equation (2). The propagation direction of SPPs wave can be dynamically controlled by changing the incident angle of excitation light. For the $\alpha =10^{\circ }$ and $\alpha =-10^{\circ }$ incident light, the propagation angle of SPPs wave increases to $\theta =9.64^{\circ }$, which is obtained from Figure 2c,d. The propagation and distribution of the SPPs field can be measured with the scanning near field optical microscope (SNOM) technique [37,38] or far-field scattered imaging system [39].

### 2.2. Nondiffracting SPPs Bessel Beam

**Figure 3:**
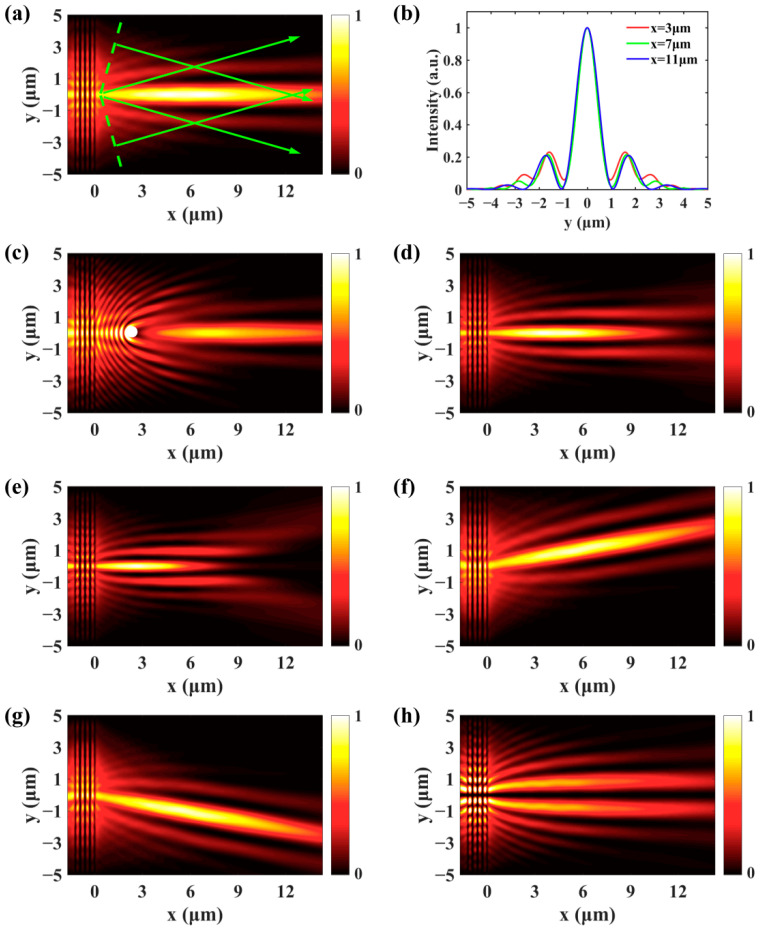
(**a**) The SPPs Bessel beam with the profile of zeroth-order Bessel function can be generated with a symmetrical phase $\beta =10^{\circ }$. (**b**,**c**) The nondiffracting and self-healing properties of the SPPs Bessel beam. By changing β, the FWHM of the main lobe and the length of the nondiffracting area can be modulated, $\beta =15^{\circ }$ (**d**) and $\beta =20^{\circ }$ (**e**). For $\alpha =10^{\circ }$ (**f**) and $\alpha =-10^{\circ }$ (**g**), the SPPs Bessel beam propagates upward and downward, respectively. (**h**) The SPPs beam takes on the profile of first-order Bessel function when the symmetrical phase is multiplied by a sign function.

In the above, the entire SPPs plane wave propagates in the same direction. However, if we encode two linear phases with different gradients on the SLM and correspondingly divide the plasmonic grating into the upper part and lower part, the propagation direction of the SPPs waves generated by these two parts will be different. The expression of the phase mask can be written as follows:

(3) $$\varphi \left(x,y\right)=-k_{0}\left|y\right|\sin\beta -k_{0}y\sin\alpha ,$$

which consists of a symmetrical phase and a linear gradient phase. For $\alpha =0^{\circ }$, the phase mask can be simplified into the symmetrical phase in Figure 1d. In this case, the SPPs wave generated by the upper part propagates downward and the lower part that excites the SPPs propagates upward. These two SPPs waves interfere constructively and the SPPs beam with the Bessel profile is generated, which can be seen from the normalized SPPs intensity distribution in Figure 3a for $\beta =10^{\circ }$. A comparison of the intensity distribution along *x* = 3 μm, *x* = 7 μm, and *x* = 11 μm is given in Figure 3b. The full-width at half-maximum (FWHM) of the main lobe is nearly the same (0.998 μm) during the propagation, which indicates that the diffraction of the SPPs Bessel beam is weak. In order to analyze the self-healing property of the SPPs Bessel beam, a metal particle with a diameter of 400 nm is placed at *x* = 2.7 μm, which is represented by the white circle in Figure 3c [28]. Around the particle, the SPPs field is seriously distorted. However, the SPPs Bessel beam recovers to its shape after the obstacle. The FWHM of the main lobe and the length of the nondiffracting area can be dynamically changed by varying the parameter β. The SPPs fields in Figure 3d,e show that the FWHM decreases and the nondiffracting area gets shorter as β increases from $15^{\circ }$ to $20^{\circ }$. The propagation direction of the SPPs Bessel beam is determined by the parameter α. For $\alpha =10^{\circ }$ and $\alpha =-10^{\circ }$, the SPPs Bessel beam propagates upward and downward, respectively, as presented in Figure 3f,g. Moreover, by adding a sign function sgn(y) to the symmetrical phase, the SPPs fields generated by the upper and lower parts of the grating are out of phase and interfere in a destructive manner. Therefore, intensity minima can be observed along the *x* axis and the profile of the SPPs beam becomes the first-order Bessel function [40], as shown in Figure 3h. In our previous study [28], the position and the profile of the SPPs Bessel beam are controlled by orthogonally linearly or circularly polarized light, which is a binary modulation. Here, the parameters α and β can be continuously modulated. Therefore, the propagation direction of the SPPs Bessel beam and FWHM of the main lobe can be arbitrarily controlled.

### 2.3. Focusing of SPPs

Furthermore, in addition to the excitation of Cherenkov SPPs wake and the generation of diffraction free SPPs Bessel beam, the plasmonic grating can function as a SPPs lens. To realize this function, the expression of the required phase mask is as follows:

(4) $$\varphi \left(x,y\right)=-k_{0}(\sqrt{f^{2}+x^{2}+y^{2}}-f)-k_{0}y\sin\alpha .$$

**Figure 4:**
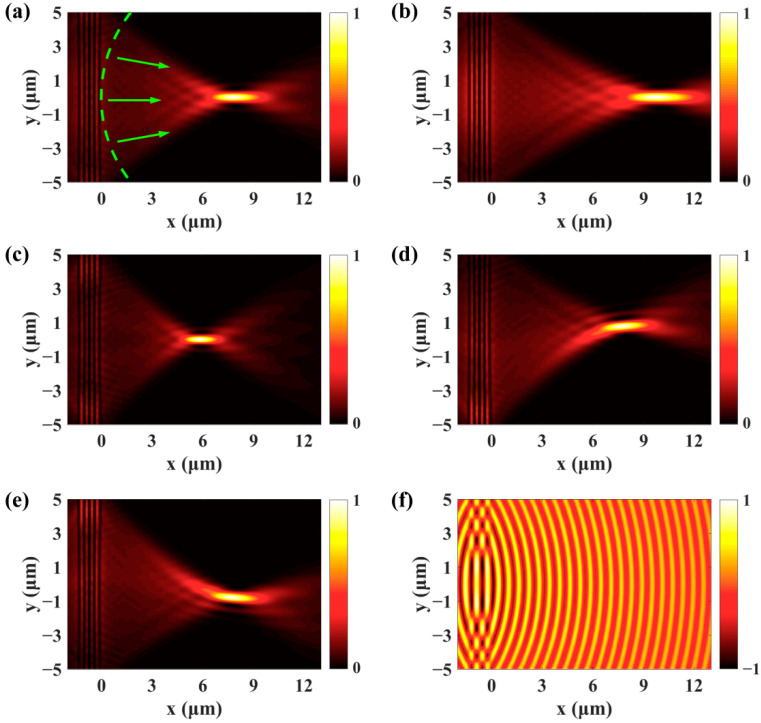
(**a**) The plasmonic grating can function as a SPPs lens by encoding a spherical phase on the SLM. (**b**–**e**) The position of the SPPs focus can be precisely controlled by changing the parameter *f* and *α*. (**f**) The polarity of the SPPs lens can be tuned from convex to concave.

The first term is a spherical phase that converges the SPPs wave to the focus. The second term is a linear gradient phase that determines the transversal displacement of the SPPs focus. For $\alpha =0^{\circ }$, the linear phase becomes zero and we only need to consider the spherical phase in Figure 1e. The normalized intensity of SPPs field is presented in Figure 4a for the *f* = 8 μm spherical phase. The SPPs wave propagates along the radial direction and the wavefront is circular. The simulated SPPs focal length is 7.85 μm, which agrees well with the setting value. By changing the focal length *f*, the SPPs wave can be focused on different positions. Figure 4b,c shows the simulated focusing of SPPs field for *f* = 10 μm and *f* = 6 μm, respectively. Moreover, if a linear gradient phase is imposed on the excited SPPs, the SPPs focus will experience a transversal displacement. As can be seen from Figure 4d,e, the SPPs focus correspondingly deviates from the center upward and downward for the $\alpha =5^{\circ }$ and $\alpha =-5^{\circ }$. The theoretical value of the displacement calculated with fsinα is 0.697 μm and the simulated value is 0.728 μm. The real parts of SPPs field in Figure 4f show that the polarity of SPPs lens can be changed from positive (convex) to negative (concave) by projecting a divergent spherical phase on the plasmonic grating. Of note, the dynamic focusing of SPPs by illuminating an arc slit with a vortex beam has been reported [41,42]. The SPPs wave can be focused on different positions by changing the topological charge. However, the alignment between the vortex beam and the arc slit is required and only the focusing function is realized. The proposed multifunctional plasmonic grating does not need the alignment and can play different roles. To experimentally demonstrate the theoretical model, the desired phase modulations can be projected to the plasmonic grating with a 4*f* system [43].

## 3. Conclusions

In conclusion, without the extra demand of fabrication, one plasmonic grating is able to play the role of three different SPPs devices by modulating the phase of excitation light. For incident light that is imprinted with a linear gradient phase, the SPPs wave propagates obliquely and the Cherenkov SPPs wake can be observed. The nondiffracting SPPs Bessel beam is generated with a symmetrical phase modulation. Moreover, the plasmonic grating can function as a SPPs lens when a spherical phase is encoded into the excitation light. Furthermore, each function can be actively controlled. The required phase can be obtained with SLM or optical devices including the wedge, axicon, and lens. Therefore, the proposed versatile approach can find applications in on-chip communications and particle manipulations.
